# The Use of Peripheral Parenteral Nutrition in Hospitalized Patients: A Clinical Case Series

**DOI:** 10.3390/healthcare14152413

**Published:** 2026-08-05

**Authors:** Alessandro Laviano, Paolo Pasquero, Emanuele Rinninella, Lidia Santarpia

**Affiliations:** 1 Department of Translational and Precision Medicine, Sapienza University, 00185 Rome, Italy; 2 Clinical Nutrition Unit, AOU Sant’Andrea, 00189 Rome, Italy; 3 Department of Medical and Specialized Sciences, A.O.U. City of Health and Science of Turin, 10126 Turin, Italy; 4 Research Center on Human Nutrition, Catholic University of the Sacred Heart, 00168 Rome, Italy; emanuele.rinninella@unicatt.it; 5 Department of Translational Medicine and Surgery, Catholic University of the Sacred Heart, 00168 Rome, Italy; 6 Clinical Nutrition Unit, Department of Medical and Surgical Sciences, Foundation “A. Gemelli University Hospital IRCCS”, 00168 Rome, Italy; 7 Internal Medicine and Clinical Nutrition Unit, Clinical Medicine and Surgery Department, Federico II University, 80131 Naples, Italy; lidia.santarpia@unina.it

**Keywords:** peripheral parenteral nutrition, dementia, anorexia nervosa, severe acute pancreatitis, malnutrition, case series, hospitalized patients

## Abstract

**Introduction:** Patients with dementia, severe gastrointestinal disorders, or anorexia nervosa are at increased risk of malnutrition and frequently require hospitalization. Nutritional management may be complicated by intolerance or refusal of enteral nutrition (EN), contraindications to EN or central parenteral nutrition (CPN), and delays in long-term strategies such as percutaneous endoscopic gastrostomy (PEG). The existing literature provides limited practical guidance for addressing these challenges in internal medicine settings. This retrospective case series describes three hospitalized adults who required prompt medical nutritional support and were managed with peripheral parenteral nutrition (PPN): one patient with dementia and acute pneumonia, one adult with anorexia nervosa unsuitable for EN, and one patient with severe acute pancreatitis. Given the scarce indications from the literature for managing these situations, the objective of this case series was to illustrate the clinical decision-making involved in these three cases rather than to demonstrate the efficacy of the PPN strategy. Cases were selected using predefined criteria to highlight therapeutic reasoning across heterogeneous conditions. **Results:** The duration of PPN administration ranged from 7 to 10 days. An initial improvement in key nutritional parameters from the first day of PPN initiation was observed after 7 days in the patient with dementia, after 1 month in the patient with anorexia nervosa, and after 8 days in the patient with severe acute pancreatitis. Overall clinical conditions improved concurrently. **Conclusions:** In these clinical contexts, PPN appeared to offer a short-term, feasible option for initiating nutritional support or serving as a temporary bridge while planning more definitive strategies, although this remains a hypothesis that requires confirmation in larger studies.

## 1. Introduction

Adult patients at risk of malnutrition are frequently encountered in internal medicine wards, particularly those with dementia, acute gastrointestinal diseases, or feeding and eating disorders [1,2,3,4,5,6,7,8]. Although these conditions differ in etiology and clinical course, they share a high risk of inadequate oral intake and rapid nutritional deterioration.

In dementia, malnutrition develops gradually due to cognitive and functional decline, dysphagia, reduced perception of hunger and thirst, and insufficient caregiving support. For this reason, routine nutritional assessment and care represent the standard recommendation for this clinical population [5,8,9].

Acute gastrointestinal diseases—and acute pancreatitis (AP) in particular—may instead precipitate an abrupt deterioration in nutritional status due to intense metabolic stress and impaired digestive function [1,10,11,12]. Severe acute pancreatitis involves systemic inflammation, hemodynamic instability, and multiorgan dysfunction, leading to high morbidity and mortality [11,12].

Anorexia nervosa (AN) is another condition frequently associated with severe malnutrition. Self-imposed dietary restriction and abnormal weight-control behaviors lead to profound nutritional deficits, while refusal of food and medical nutrition often complicates inpatient management [13,14,15]. In addition, recovery from AN may require several years [16].

Despite this evidence, practical recommendations tailored to internal medicine settings remain limited, particularly when conventional nutritional strategies cannot be implemented. Enteral nutrition (EN) and central parenteral nutrition (CPN) are generally preferred for hospitalized patients with these conditions, but may be contraindicated, not tolerated, or not feasible [1,5,6,13,14,15]. In such circumstances, peripheral parenteral nutrition (PPN) might represent a pragmatic option when EN or CPN cannot be promptly initiated [6,15,17,18,19]. In this context, PPN may support early metabolic stabilization and potentially serve as a temporary bridge toward individualized long-term nutritional plans, provided that its suitability is carefully assessed.

In fact, low-osmolality formulations are required, inherently involving high-volume infusion. Therefore, patient suitability for this type of administration, in addition to the adequacy of peripheral veins, should be carefully assessed, applying best practices for the choice and use of the peripheral venous access device [6,18,19]. Short-term administration is also recommended [6,18,19]. All these considerations sensibly reduce the risk of thrombophlebitis, a frequently reported complication of PPN [6,18,19].

Notably, as generally recommended for all patients undergoing rapid refeeding, refeeding syndrome should be carefully prevented, particularly in individuals with severe anorexia nervosa, who are especially vulnerable to this potentially life-threatening complication associated with marked fluid and electrolyte imbalances [20].

On this basis, as a group of clinicians (the clinical group is composed of A. Laviano, P. Pasquero, E. Rinninella, and L. Santarpia, all authors of this paper) focusing on the implementation of PPN in Italian internal medicine settings, we present a retrospective case series of hospitalized patients who required urgent nutritional support but exhibited distinct clinical characteristics: one with severe dementia, a second with AN, and a third with severe acute pancreatitis.

The objective of this retrospective case series was to illustrate the clinical decision-making involved in these three cases rather than to demonstrate the efficacy of the PPN nutritional strategy.

The selection of clinical cases was based on the following predefined criteria: a history of hospitalization in an Internal Medicine Department in the preceding two years; diagnosis of malnutrition with concurrent dementia, anorexia nervosa, or acute pancreatitis; urgent need for medical nutrition; and contraindication, intolerance, infeasibility, or refusal of enteral or central parenteral nutrition.

A total of 14 patients met the eligibility criteria, and three cases were selectively included. These three cases were chosen among all eligible patients because they were clinically suitable for the administration of PPN and exemplify clinical situations in which the implementation of artificial nutrition may be hindered by contextual difficulties encountered in real-world hospital practice. Such difficulties included administrative constraints, such as delays in percutaneous endoscopic gastrostomy (PEG), the presence of underlying or concomitant serious diseases requiring urgent medical care in addition to nutritional support, and complex psychological features that challenge clinical management. The three cases included in the case series represented these conditions.

## 2. Case Presentations

### 2.1. Case 1: Nutritional Strategy in Patient Affected by Severe Dementia

**Figure 1:**
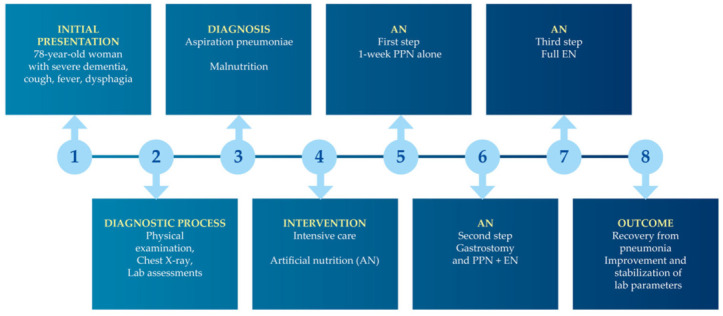
Case 1 summary flow chart.

Figure 1 illustrates the overall workflow of the case presentation.

#### 2.1.1. Case Presentation

A 78-year-old woman with a history of long-standing hypertension, hypercholesterolemia, osteoporosis, and an esophageal injury resulting in a stable esophageal stricture presented to the Department of Internal Medicine at an Italian university hospital with dyspnea, productive cough, and fever of 48 h duration. Over the previous months, she had experienced progressive cognitive decline evolving into severe dementia, accompanied by dysphagia, reduced oral intake, recurrent coughing during meals, and a 5% weight loss in the last month. In the days preceding admission, fever led to lethargy and somnolence, and oral feeding was administered by syringe to maintain caloric intake. The patient received home care provided by family members.

In the days preceding admission, fever led to lethargy and somnolence, and oral feeding was administered by syringes to maintain caloric intake.

At the time of admission, the patient was taking proton pump inhibitors. Her past medical history included long-standing arterial hypertension for over 20 years, treated with ACE inhibitors; hypercholesterolemia previously managed with statin therapy, discontinued due to myalgias; and diffuse osteoarthritis. However, due to her advanced age and overall clinical condition, some difficulties arose in collecting family history.

On admission, she appeared febrile (38.2 °C), tachypneic (26 breaths/min), hypoxemic (SpO_2_ 90%), and dehydrated, with relative hypotension (100/60 mmHg) and acute confusion.

Chest auscultation revealed diffuse bilateral rhonchi and crackles, more pronounced in the right mid-basal lung fields. Chest radiography showed ground-glass opacities and consolidation in the right lower lobe, consistent with aspiration pneumonia. In keeping with this, laboratory tests demonstrated leukocytosis and elevated inflammatory markers (Table 1).

Physical nutritional assessment revealed a body weight of 46 kg, a height of 162 cm, and a BMI of 17.56 kg/m^2^. In addition, the patient met the GLIM criteria for malnutrition, presenting two phenotypic criteria (weight loss, low BMI) and three etiologic criteria (low food consumption, disease burden, and inflammation), as indicated by elevated C-reactive protein (CRP), confirming a state of protein–energy malnutrition. This diagnosis was further supported by laboratory abnormalities (Table 1).

Following the diagnosis of aspiration pneumonia and evidence of dehydration, appropriate antibiotic therapy and intravenous hydration were initiated to stabilize the patient’s clinical condition. Given the evident state of protein–energy malnutrition, medical nutrition support was deemed necessary.

However, oral feeding was contraindicated due to the high risk of aspiration, and nasogastric tube placement was not feasible due to esophageal stenosis. Consequently, EN was identified as the most appropriate long-term strategy. Nevertheless, several days were needed to achieve clinical stabilization, complete the pre-procedural evaluations, and proceed with gastrostomy for the administration of EN. To prevent further nutritional deterioration during this interval, PPN was initiated.

Based on estimated caloric and protein requirements, and the need for a moderately hypotonic formulation suitable for PPN administration, the patient received a pre-formulated preparation of approximately 1500 mL containing 100 g of glucose, 46 g of amino acids, and fourth-generation lipids, providing a total of 1000 kcal/day. Supplementation with micronutrients, such as trace elements and vitamins, was associated with PPN administration. The peripheral venous catheter was replaced every 72–96 h to minimize the risk of phlebitis.

After seven days of PPN and appropriate antibiotic therapy for aspiration pneumonia, the patient showed a correction of dehydration and hydroelectrolyte imbalances, recovery of the blood cell count, normalization of inflammatory markers, and improvement in metabolic–nutritional parameters. These changes resulted in clinical conditions suitable for PEG and the initiation of EN, with the resolution of pneumonia.

The transition to EN was initiated progressively, thereby minimizing the risk of complications. EN was then introduced gradually after PEG, with a short mixed-feeding phase, and reached full EN within three days.

The initiation of EN and the achievement of the full EN regimen led to the sustained stabilization of all laboratory parameters and to an improvement in the patient’s nutritional status. Seven days after PEG, the patient was discharged and returned for the follow-up visit two weeks after the procedure.

Given her advanced stage of dementia, the patient had a limited ability to express her perceptions regarding the treatment received. Nevertheless, no adverse events or signs of intolerance to the nutritional treatments were observed.

Protocols for PPN and EN administration are reported separately in Table 2 and Table 3.

Table 4 summarizes the sequence of the nutritional treatment.

**Table 4:** Sequence of the nutritional treatment.

1	2	3
7 days of exclusive PPN	PEG 3 days of mixed-feeding PPN + EN gradually increased	Full EN

Abbreviations: PPN, peripheral parenteral nutrition; PEG, percutaneous endoscopic gastrostomy; EN, enteral nutrition. Figure 2 and Figure 3 illustrate the overall trends of the key inflammatory and nutritional laboratory parameters.

**Figure 2:**
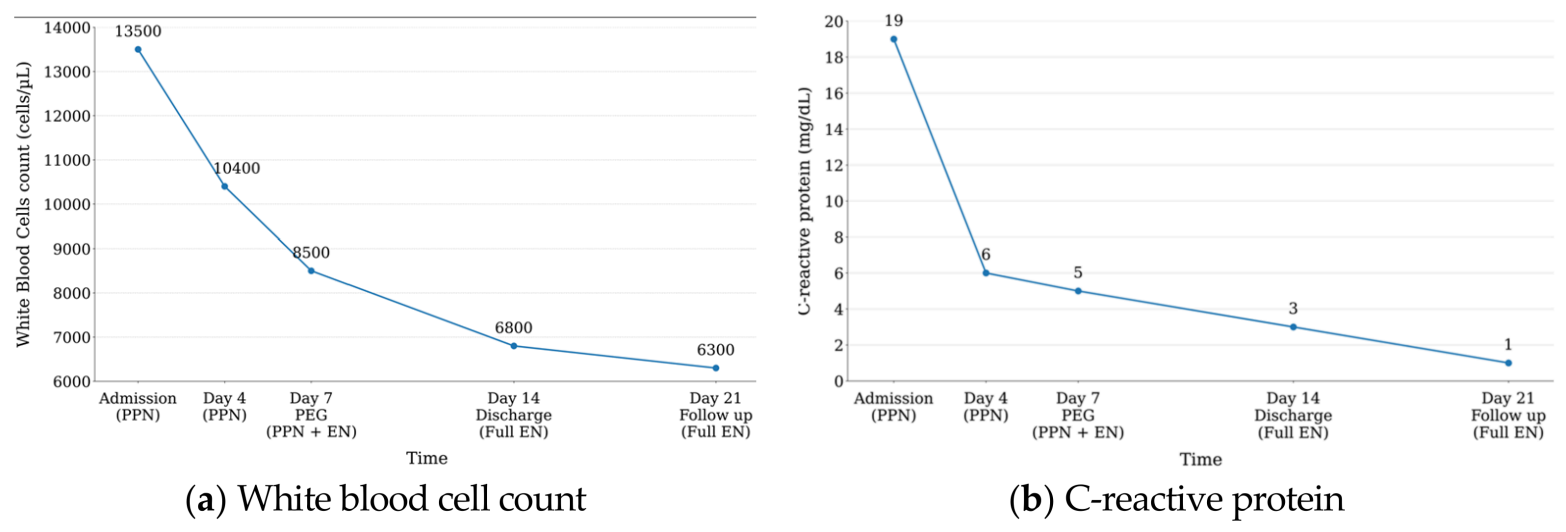
Key inflammatory laboratory parameters.

**Figure 3:**
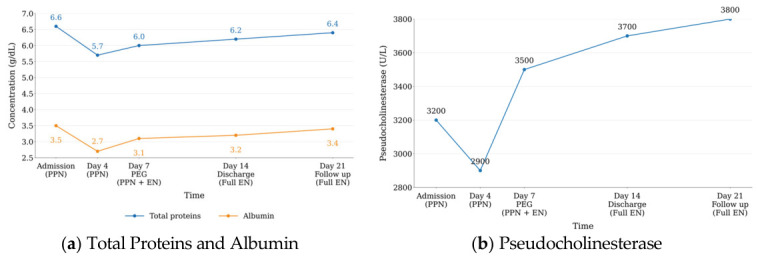
Key nutritional laboratory parameters.

#### 2.1.2. Discussion

Individuals with severe dementia are highly prone to malnutrition due to reduced sensitivity to hunger and thirst, apraxia, visuospatial impairment, and dysphagia [5,8]. Social factors—including the lack of skilled caregivers, comorbidities, and adverse drug effects—further contribute to nutritional decline [5,6]. Malnutrition, in turn, may accelerate cognitive decline through nutrient deficiencies and dehydration, creating a cycle that further reduces oral intake [5].

In hospitalized patients with advanced dementia, early nutritional intervention is therefore essential, with oral supplementation representing the preferred first-line approach [5,8]. However, cognitive and motor impairment, anorexia, and swallowing difficulties often limit adequate intake, making EN or parenteral nutrition (PN) necessary in selected cases [5]. Nevertheless, while EN has been more extensively studied, evidence regarding parenteral nutrition in this population remains limited, and standardized protocols are lacking [5].

Consequently, nutritional strategies should be individualized, carefully weighing indications, contraindications, and the benefit–risk profile.

In this case, the patient presented with aspiration pneumonia and overt malnutrition based on clinical and laboratory findings. Although treatment of the respiratory infection was a priority, nutritional rehabilitation also required prompt attention because the two conditions may exacerbate one another. Oral feeding was contraindicated due to the risk of aspiration, and nasogastric tube placement was not feasible because of esophageal stenosis. EN remained the most appropriate long-term option; however, procedural delays risked further nutritional deterioration. For this reason, PPN was initiated as a temporary measure.

The subsequent clinical course, including an improvement in hydroelectrolyte imbalance, may reflect the combined effect of metabolic stabilization achieved through nutritional support and the concurrent medical therapies, with antibiotic treatment playing a central role in addressing the underlying pneumonia.

From a practical clinical standpoint, PPN provided minimal nutritional intake until gastrostomy placement, acting as a short-term bridge to definitive enteral nutrition. EN remains the preferred modality when oral intake is not feasible, and PPN should be considered only when indicated and for limited durations.

#### 2.1.3. Conclusions

The use of PPN in patients with dementia and associated malnutrition might represent a feasible bridge toward a long-term nutritional strategy also suitable in the post-discharge phase. Achieving this objective is crucial, as improving nutritional status in older patients with dementia is essential to reducing morbidity and mortality risk.

### 2.2. Case 2: Nutritional Support in a Malnourished Patient with a History of Anorexia Nervosa

**Figure 4:**
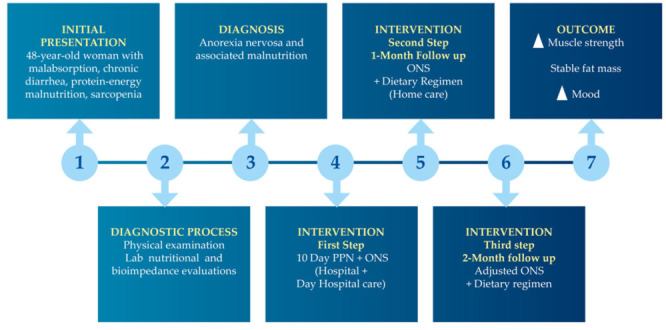
Case 2 summary flow chart.

Figure 4 presents a comprehensive schematic summary of the case.

#### 2.2.1. Case Presentation

A 48-year-old woman was admitted to the Internal Medicine ward of an Italian university hospital for malabsorption with chronic diarrhea (up to 10 episodes/day), protein–energy malnutrition, and sarcopenia, in the context of an anxiety–depressive disorder with prominent somatization. Her symptoms included edema, headache, peripheral vasoconstriction, nausea, hypotension, and alternating episodes of diarrhea and constipation. 

Her personal history included long-standing anxiety, episodes of compromised health during childhood, and recurrent reactive hypoglycemia following significant weight loss related to prolonged occupational stress in recent years. She also reported previous dietary patterns characterized by a high glycemic load and associated gastrointestinal imbalance.

Her family history was notable for maternal chronic pain and major depression leading to prolonged hospitalization.

Her past medical history included a previously resolved but not documented eating disorder associated with emotional distress, sleep disturbances during adolescence, insulin resistance, food intolerances, autoimmune markers, pelvic floor dysfunction, and a stable pituitary microadenoma. In the year preceding admission, she had experienced marked weight loss with severe malnutrition, during which parenteral nutrition had been considered due to intolerance to nasogastric feeding. She reported multiple allergies, including contrast media, isothiazolinones, sodium metabisulfite, paracetamol, low-dose aspirin, and betamethasone. At the time of evaluation, she was employed in the educational sector and was in a stable long-term relationship.

At admission, the patient was alert, cooperative, and fully oriented, though markedly underweight. The clinical examination, nutritional parameters, bioelectrical impedance analysis results, and abnormal laboratory findings at admission are summarized in Table 5, Table 6 and Table 7.

A nutritional risk was highlighted through nutritional screening tools (NRS-2002 = 4, MUST = 2), bioelectrical impedance analysis, blood chemistry, and a CONUT score of 2. A diagnosis of malnutrition was confirmed based on the GLIM Criteria.

The combined clinical evaluation—including physical examination, laboratory tests, nutritional assessment, bioelectrical impedance analysis, family history, and a previously reported but undocumented episode of anorexia nervosa—together with the marked weight loss and severe malnutrition observed in the preceding year, supported the diagnosis of a persistent eating disorder. Although the patient reported resolution of the disorder, the observed clinical parameters indicated its persistence. This interpretation was reinforced by somatic features typically associated with anorexia nervosa, such as bradycardia with hypotension, alternating bowel function, mild pitting edema due to hypoalbuminemia, and elevated amylase and ALT levels suggestive of recurrent vomiting and nutrition-related hepatic dysfunction.

Given her compromised nutritional and overall health status, an immediate nutritional strategy was implemented, and the patient was concurrently referred to the psychiatrist of the local multidisciplinary team. As she declined the psychiatric assessment, reporting ongoing care by a private specialist, the intervention was deferred to a later stage of follow-up to maintain a constructive therapeutic relationship and support adherence.

According to the calculated energy and protein requirements, the patient received a pre-formulated 1500 PPN preparation containing 100 g of glucose, 46 g of amino acids, and a fourth-generation lipid emulsion in a low-osmolar formulation.

The infusion was administered through a peripheral venous catheter under regular clinical supervision. Given the high risk of refeeding syndrome, PPN was started at approximately 50% of the target daily volume and progressively increased based on clinical and biochemical monitoring. Micronutrient supplementation, including vitamins and trace elements, was provided concomitantly with the PPN regimen according to institutional clinical practice.

Oral nutritional supplementation (ONS) was introduced concomitantly with PPN using two bottles of a high-energy (2.4 kcal/mL), high-protein formula, each providing 300 kcal and 20 g of protein. Intake was progressively advanced according to the patient’s clinical tolerance and oral intake, complementing rather than replacing the individualized PPN regimen.

Table 8 illustrates the PPN protocol.

Due to the patient’s severe malnutrition (BMI 14.2 kg/m^2^), careful monitoring for refeeding syndrome was implemented. Serum phosphate, magnesium, potassium, glucose levels, hydration status, and vital signs were regularly assessed throughout the nutritional intervention. Thiamine and micronutrient supplementation were administered prophylactically, and no evidence of refeeding syndrome was observed. The intervention was well-tolerated.

The patient was discharged under a protected care regimen and admitted to the Day Hospital after five days to continue parenteral nutrition for an additional five days per week, totaling ten days of supplemental parenteral nutrition. The entire nutritional regimen was well-tolerated.

At the one-month follow-up, she reported persistent eructation, lip paresthesia, bitter taste, headache, and abdominal distension, although she appeared more emotionally stable and resolute in her intention to resume the therapeutic course. Despite improvements in muscular strength, protein–energy malnutrition persisted; therefore, a personalized dietary plan was introduced continuing the already administered oral nutritional supplementation (ONS).

At the two-month follow-up, the patient continued to experience gaseous colic, digestive discomfort with marked eructation, constipation, and postprandial epigastric constriction, along with bilateral pretibial edema.

Notably, the patient indicated acceptance of the entire therapeutic nutritional plan, reporting no signs of intolerance or related adverse events.

Table 9 summarizes the sequence of the nutritional treatment.

**Figure 5:**
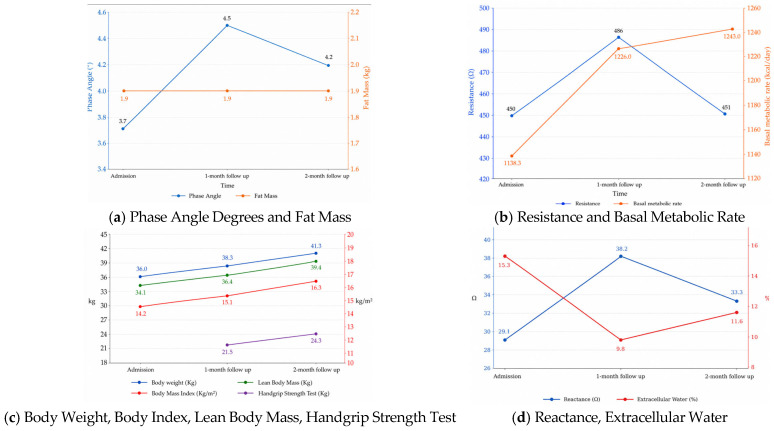
Overall trends of the nutritional and bioelectrical impedance parameters.

Updated clinical and nutritional assessments demonstrated progressive improvement in muscle strength with stable fat mass. The overall trend of the nutritional and bioelectrical impedance parameters is illustrated in Figure 5a–d.

#### 2.2.2. Discussion

The prevalence of AN is increasing, affecting 1.4% of women and 0.2% of men, and accounting for the highest mortality among psychiatric conditions [13]. The standardized mortality rate ranges from 5.6 to 15.9 in the most severe cases, with death predominantly caused by organic sequelae and fatal self-harm [13].

Epidemiological data also indicate that, in the past, the onset of AN was predominantly observed in adolescents and young adults, with a higher prevalence among females. However, it is now increasingly diagnosed both in childhood and in individuals over 40 years of age [21]. Furthermore, the incidence among males is rising, partly due to greater efforts to identify and diagnose the disorder in this population [22].

An additional noteworthy finding is that, despite the fact that almost a quarter of patients fully recover after diagnosis and treatment, a high rate of relapse may still occur during follow-up, requiring intensive and multidisciplinary intervention to restore and maintain an acceptable weight, normalize eating behaviors, and manage potential complications [16,21,23,24].

The treatment of severely malnourished patients generally includes the administration of EN; once physical stabilization is achieved, psychiatric and/or psychotherapeutic interventions may be implemented [13]. Indeed, these patients, due to a persistent and distorted perception of body image, not only exhibit marked resistance to oral food intake but also frequently refuse EN via a nasogastric tube, even in the presence of severe malnutrition and clinically concerning conditions [6,15,21].

Nonetheless, weight gain has been proved to improve cognitive and behavioral symptoms and enhance the efficacy of psychological and pharmacological treatments [21]. Consequently, when patients do not tolerate or are not compliant with EN, it becomes necessary to identify a viable alternative nutritional strategy.

Therefore, the choice of nutritional approach should be individualized based on the care setting—such as hospitalization, day hospital, or home care—as well as the patient’s clinical condition and personal characteristics [21]. In all cases, particular attention must be paid to preventing refeeding syndrome in severely malnourished patients [20,21].

In addition to these considerations, although parenteral nutrition is generally not recommended as a first-line approach in patients with AN, due to the heightened risk of infections and metabolic complications, the use of PPN may represent a timely nutritional intervention capable of progressively supporting the transition toward full oral intake [6,13,15]. However, there is concordance that central parenteral nutrition is indicated when severe electrolyte disturbances are present, when fluid administration must be strictly limited, or more broadly, when the patient’s clinical condition is critically compromised [6].

This clinical case concerns a 48-year-old woman who reported a past recovery from anorexia nervosa, although no documentation was available to substantiate either the previous diagnosis or the remission. Her past medical history included recurrent emotional difficulties and digestive disturbances that were still present at admission. Furthermore, the patient exhibited somatic abnormalities characteristic of anorexia nervosa [13,14]. Laboratory abnormalities included elevated amylase and ALT levels that may reflect recurrent vomiting and nutrition-related hepatic dysfunction, respectively, the latter linked to increased autophagy and depletion of hepatocellular glycogen stores during prolonged caloric deprivation [13]. All these elements—considered together with the nutritional findings, results from the bioelectrical impedance analysis, and information on past family dynamics—raised concern for the persistence or recurrence of anorexia nervosa, warranting continued psychiatric evaluation and multidisciplinary management.

The condition of malnutrition was identified through specific assessment tools—including a CONUT score of 2, an NRS-2002 score of 4, and a MUST score of 2—and was subsequently validated using the GLIM Criteria, which are currently regarded as the gold standard for establishing the diagnosis of malnutrition [25,26].

With regard to the psychological framework, the patient refused referral to the psychiatrist of the local multidisciplinary team, as she was already being treated by a private psychiatrist, and explicitly requested that her specialist not be involved—a circumstance still frequently encountered in clinical practice. Consequently, psychiatric assessment was deferred to a later phase of follow-up to safeguard an effective therapeutic alliance and promote adherence.

This scenario, in which the patient reports notable past events that are not documented and also refuses specialist interventions, represents a common challenge in clinical practice. It requires careful assessment to ensure an appropriate accommodation of the patient’s attitude and expressed willingness, and to accurately interpret and contextualize symptoms together with the reported medical history in order to define the most suitable care approach.

Consequently, given that the restoration of nutritional status and weight gain may contribute to improving cognitive disturbances in patients with AN—an aspect that typically requires the longest recovery time—it was deemed necessary to promptly initiate hospital-based nutritional therapy through a combination of PPN and ONS.

This approach was selected due to prior intolerance to EN and was facilitated by the patient’s cooperative behavior.

Over time, the nutritional intervention was continuously tailored to the patient’s nutritional status, bioimpedance parameters, and clinical setting—hospital, day hospital, and home care—progressively transitioning from supplementary PPN combined with ONS to ONS alone alongside an oral diet.

Overall, the patient exhibited gradual improvements in nutritional and functional parameters. Concurrently, clinical follow-up suggested improved emotional stability and greater adherence to the nutritional program; however, no formal psychiatric outcome measures were collected, and no causal relationship between nutritional support and psychological improvement can be inferred.

#### 2.2.3. Conclusions

This case suggests that, in a patient with AN who is intolerant to enteral nutrition but remains cooperative, the early use of PPN combined with oral nutritional supplements may have facilitated the subsequent transition to adequate oral intake. This occurred without the need for continued parenteral support, although no correlation can be established between nutritional support and psychological improvement. Notably, the management of patients with AN should not rely solely on medical nutrition, but on the coordinated work of a multidisciplinary team, including psychiatrists.

### 2.3. Case 3. Nutritional Management in Severe Acute Pancreatitis

**Figure 6:**
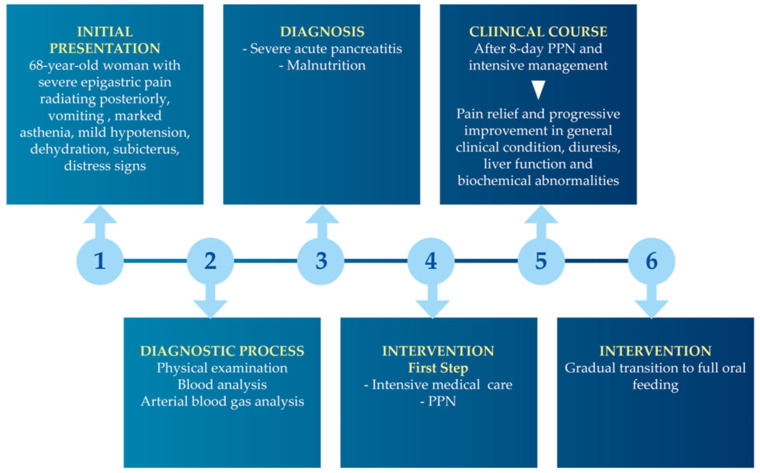
Case 3 summary flow chart.

Figure 6 provides a schematic overview of the entire case presentation.

#### 2.3.1. Case Presentation

A 68-year-old woman presented to the Emergency Medicine Department of an Italian university hospital with severe epigastric pain radiating posteriorly, accompanied by vomiting and marked asthenia. She was subsequently transitioned to the Internal Medicine Department of the same institution. Her recent history was notable for alcohol-related chronic pancreatitis and pharmacologically treated hypertension. She reported a 7% unintentional weight loss over the preceding month, with a BMI of 21.4 kg/m^2^.

Her past medical history included the onset of menopause in her early fifties, followed by the development of hypertension, along with a long-standing pattern of unstructured eating habits. She also reported a prolonged history of daily alcohol consumption beginning in late adolescence, generally involving moderate intake with meals and additional consumption during evenings and weekends.

Family history was significant for hypertension and hypercholesterolemia on both the maternal and paternal sides, with no reported cases of diabetes or neoplastic disease.

On clinical examination, the patient was alert and oriented, with a Glasgow Coma Scale (GCS) score of 15, although she appeared to be in significant distress and was febrile at presentation. Physical findings included subicterus and signs of dehydration, along with mild hypotension secondary to hypovolemia. Arterial blood gas analysis revealed mild hypoxemia on room air, associated with mild metabolic acidosis. The APACHE II score was calculated at 20. The laboratory findings are summarized in Table 10.

In addition, the patient met the GLIM criteria for malnutrition, presenting one phenotypic criterion (weight loss) and three etiologic criteria (low food consumption, disease burden, and inflammation), as indicated by elevated CRP.

The integration of clinical findings, nutritional assessments, laboratory abnormalities, and the APACHE II score indicated overt malnutrition and supported the suspicion of severe acute pancreatitis (SAP) with a high risk of systemic complications.

These features warranted the initiation of nutritional support and close metabolic monitoring.

Intensive medical care, including antibiotics, analgesics, and correction of electrolyte imbalances, was undertaken.

Due to severe abdominal pain and the resulting technical difficulty in placing a nasojejunal tube, EN was not feasible. PPN was therefore selected as the nutritional strategy, while placement of a central venous catheter was excluded because of the increased risk of hemorrhagic complications associated with thrombocytopenia and prolonged INR. The PPN infusion consisted of a 1904 mL mixture providing 135 g of glucose, 60 g of amino acids (9.8 g of nitrogen), 54 g of lipids, and electrolytes (Na 48 mmol, K 36 mmol, Mg 6.0 mmol, Ca 3.0 mmol, P 15.6 mmol), for a total of 1300 kcal (1100 kcal non-protein) and an osmolarity of 850 mOsm/L. The lipid component consisted of 30% soy oil, 30% medium-chain triglycerides, 25% olive oil, and 15% fish oil, providing ω-3 fatty acids and yielding an n-6:n-3 ratio of 2.5:1.

The PPN infusion was supplemented with vitamins and trace elements, with particular attention to fluid and electrolyte administration and management to prevent fluid overload. Concurrently, glycemia, liver and kidney function, inflammatory markers, and signs of infection were closely monitored.

Table 11 illustrates the PPN composition and management.

The patient showed good tolerability for PPN, with progressive improvements in general condition, diuresis, and liver function. After 8 days of PPN infusion and intensive medical care, her body temperature normalized, accompanied by pain relief, an increase in albumin levels to 3.0 g/dL, and stabilization of the coagulation profile.

Consequently, the patient was gradually transitioned to oral feeding, starting with a low-lipid diet divided into small meals, while maintaining supplementary parenteral support for the first 3 days as a bridge to full oral nutrition (ON). The patient reported tolerability and satisfaction following the restoration of full oral nutrition within 11 days of treatment initiation. No adverse events associated with the nutritional plan were observed.

Table 12 summarizes the sequence of the nutritional treatment.

The trends of the laboratory findings during hospitalization are reported in Table 13, Table 14, Table 15 and Table 16.

**Figure 7:**
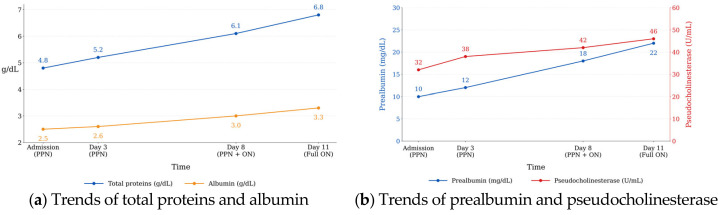
Trends of nutritional blood parameters.

Figure 7 illustrates the trends of the nutritional blood parameters.

#### 2.3.2. Discussion

Severe acute pancreatitis (SAP) is a potentially life-threatening condition frequently associated with profound metabolic and systemic complications and may require surgical intervention [10,11,12].

Malnutrition is a common concern in patients with SAP, and appropriate nutritional support may contribute to reducing mortality and infectious complications [10].

As a matter of fact, in acute pancreatitis (AP), the inflammatory response and the metabolic impact of infectious complications substantially increase energy expenditure, leading to significant protein catabolism, electrolyte disturbances, acid–base imbalance, and reductions in vitamin and micronutrient levels [10].

In addition, glucose metabolism is markedly affected, because inflammatory injury to pancreatic islet cells may reduce insulin secretion, while transient insulin resistance is frequently described [10,12].

Evidence also suggests that stress-related hyperglycemia may be exacerbated by increased production of glucocorticoids, catecholamines, and glucagon [12].

Taken together, current evidence highlights that nutritional intervention represents a key component of the medical management of patients with SAP and of those at risk of progressing to severe disease [10].

In this context, management requires a multimodal approach, including hemodynamic stabilization, the correction of electrolyte and metabolic abnormalities, and the timely provision of adequate nutritional support [12].

In this regard, EN should be considered the first-line nutritional strategy in SAP, as it has demonstrated greater safety and efficacy than PN in reducing mortality, infectious complications, the need for surgical interventions, and the length of hospitalization [1]. Nevertheless, when EN is contraindicated or not tolerated, PN may be considered, following a careful evaluation of risks and benefits [1,6].

This case report illustrates the clinical course of a patient with SAP and highlights the role of PPN in supporting recovery when EN and central venous access are contraindicated.

The patient was admitted with a diagnosis of SAP. Given the severity of her clinical presentation, intensive medical management was initiated, targeting the stabilization of vital signs, the correction of electrolyte and fluid imbalances, infection control, and pain management.

With regard to the nutritional intervention, EN was contraindicated due to persistent abdominal symptoms, while central parenteral nutrition was excluded because of the patient’s thrombocytopenia, elevated INR, and the associated risk of hemorrhage.

Consequently, PPN was employed as a feasible alternative, providing adequate metabolic support to concurrent intensive care, with rapid and sustained normalization of laboratory parameters and maintenance of nutritional status, contributing to the patient’s progressive recovery and the gradual, successful transition to oral feeding.

#### 2.3.3. Conclusions

This case suggests that, in SAP, when EN is not viable and central venous access is contraindicated, PPN might represent a feasible temporary option as a supplement to intensive medical treatment, provided that rigorous clinical and laboratory monitoring is maintained.

The favorable outcome observed in this patient is compatible with the hypothesis that PPN could be considered within the nutritional management strategies for SAP under specific clinical constraints, while acknowledging that further studies are needed to clarify its role and generalizability.

## 3. Overall Discussion

Malnutrition that is present at hospital admission or develops during hospitalization is associated with poorer clinical outcomes, a prolonged length of stay, and increased healthcare costs, predisposing hospitalized patients to nosocomial infections and thereby contributing to higher morbidity and mortality [27,28].

Taken together, these findings highlight the importance of early nutritional assessment and regular monitoring throughout hospitalization to ensure the timely initiation of appropriate nutritional support [27].

In line with this evidence, the three cases presented in this series illustrate real-world clinical scenarios in which hospitalized patients exhibited significant nutritional risk and, due to specific contraindications, intolerance, or clinical infeasibility, could not receive EN or CPN—interventions that are respectively considered the first-line option and the most appropriate approach in critical conditions [1,5,13,15]. For more details on the cases reported, see the Supporting Information.

In the patient with severe dementia, PPN was administered as a metabolic bridge to full enteral nutrition while awaiting PEG placement, leading us to hypothesize that PPN could represent a pragmatic short-term option for mitigating acute caloric deficits without resorting to more invasive central venous access.

Similarly, observation of the clinical case concerning the patient with anorexia nervosa suggests that PPN might have contributed to early nutritional improvement and clinical stabilization. Indeed, the hospital management of severe anorexia is frequently complicated by the refusal of food or standard medical nutrition. Under such circumstances, PPN may offer a less invasive and potentially better-tolerated alternative nutritional strategy during the acute phase of refeeding, possibly reducing the psychological impact often associated with nasogastric tubes.

With regard to the case of severe acute pancreatitis, the available literature indicates that SAP triggers intense systemic inflammation and high metabolic demands, thus requiring intensive medical care and prompt nutritional intervention [10]. In this setting, EN is recommended as the first option, whereas parenteral nutrition is considered when EN may not be tolerated due to prolonged ileus or abdominal pain, may be insufficient to meet nutritional requirements, or is contraindicated [1,6,10,12].

In this patient, PPN was used as a supplement to intensive medical care in an attempt to prevent further nutritional and clinical deterioration, because CPN was excluded due to thrombocytopenia, elevated INR, and the associated risk of hemorrhage.

Nevertheless, in all these conditions, PPN should not be regarded as the primary therapeutic approach to recovery but rather as foundational support for concurrent medical treatments.

Considered together, these examples may suggest that PPN could represent a pragmatic and feasible short-term option in carefully selected cases of hospitalized patients when first-line nutritional strategies cannot be promptly initiated.

This interpretation is consistent with the recent literature describing PPN as a potential temporary bridge toward individualized long-term nutritional plans, provided that careful assessment of patients’ suitability and adherence to best clinical practice for venous access are ensured [6,18]. Furthermore, it should be acknowledged that PPN is not feasible in the home setting, thus necessitating hospitalization or day hospital care.

Additionally, attention to micronutrient provision remains essential when prescribing PPN because pre-formulated multichamber bags do not contain all necessary micronutrients; additional tailored supplementation is required to ensure adequate intake [29,30].

Finally, some studies suggest that PN-delivered ω-3 fatty acids may contribute to modulating the response, possibly supporting infection control and liver recovery [31]. In particular, patients with SAP and multiple organ failure have been described as possibly benefiting from ω-3-enriched PN, with a potential association with better respiratory function and shorter continuous renal replacement therapy compared with standard care [31]. Nevertheless, well-designed studies will be required to substantiate this observation and determine its broader applicability [31]. In this context, parenteral ω-3 supplementation has been mentioned as a possible—and currently highly uncertain—alternative to enteral administration, a notion that remains purely exploratory and requires validation through further rigorous research [31].

Overall, the observations from our clinical cases should be viewed as descriptive and hypothesis-generating, providing preliminary insights for future research rather than confirmatory evidence.

Strengths: This case series presents concrete examples of real-world management in hospitalized patients with complex clinical or behavioral conditions requiring short-term nutritional support. These cases highlight practical situations that are not yet fully addressed in the literature. They also describe circumstances in which peripheral parenteral nutrition was considered as a pragmatic temporary option when enteral or central parenteral routes were not immediately feasible.

Limitations: The findings of this case series must be interpreted in light of several limitations. The small sample size, the clinical heterogeneity of the patient population and the absence of a comparator group do not allow for the direct inference of PPN clinical efficacy or the generalization of these results to broader populations. The retrospective nature of this case series and the real-world clinical management of patients did not allow the retrieval of all conventional and recognized diagnostic data. The literature collection provided essential clinical context for the three cases described, without involving a structured literature search, which may have limited the completeness of the contextual evidence. As a retrospective case series, the positive clinical outcomes observed may be subject to selection or publication bias.

## 4. Concluding Remarks

- Hospitalization is frequently associated with a decline in patients’ nutritional status.
- This small case series suggests that peripheral parenteral nutrition might represent a feasible short-term nutritional support option in three distinct clinical scenarios—dementia, anorexia nervosa, and severe acute pancreatitis—when other strategies are temporarily not applicable.
- Within this context, PPN might serve as a temporary bridge while planning longer-term nutritional strategies or awaiting the recovery of spontaneous oral intake, but findings from this case series cannot yet be generalized.
- Further large clinical studies are needed to clarify the effectiveness and safety of PPN in these patient categories, and future guidelines should better address such complex or atypical conditions.

## Figures and Tables

**Table 1:** Laboratory abnormalities upon admission.

Parameter	Values	Normal Range
WBC (cells/µL)	13,500	4000–10,000
Pseudocholinesterase (U/L)	3200	5300–12,900
Urea (mg/dL)	54	16.6–48.5
Creatinine (mg/dL)	1.56	0.5–1.0
Uric acid (mg/dL)	7.2	2.4–5.7
Sodium (mmol/L)	148	136–148
Potassium (mmol/L)	3.4	3.5–5.1
Ca (mg/dL)	8.1	8.60–10.0
C-Reactive Protein (mg/dL)	19	<0.5
Total proteins (g/dL)	6.6	6.4–8.3
Albumin (g/dL)	3.5	3.5–5.2

Abbreviations. WBC, white blood cells.

**Table 2:** Peripheral parenteral nutrition protocol.

Parameter	Value
Body weight	60.7 kg
Body Mass Index	21.4 kg/m^2^
Indication for PPN	Abdominal pain. Acute pancreatitis. Technical difficulties in placing nasojejunal tube
PPN formulation	SMOF 1904 mL
Energy provided (full regimen)	1300 kcal
Amino acids	68 g
Carbohydrate	135 g
Total infusion volume	1904 mL
Initial infusion volume	
Osmolarity	850 mosm/L
Lipid formulation	54 gr (30% soy oil, 30% medium-chain triglycerides, 25% olive oil, and 15% fish oil) providing ω-3 fatty acids and yielding an n-6:n-3 ratio of 2.5:1.
Electrolytes	Na 48 mmol, K 36 mmol, Mg 6.0 mmol, Ca 3.6 mmol, P 14.5 mmol
Micronutrient supplementation	Cr 0.02 μmol, Cu 20 μmol, Fe 20 μmol, Mn 5 μmol, I 1 μmol, Fl 50 μmol, Mo 0.2 μmol, Se 0.6 μmol,
	Zn 100 μmol.
	vitamin A 3500 IU, vitamin D3 220 IU, vitamin E 11.2 IU, vitamin C 125 mg, vitamin B1 3.51 mg, vitamin B2 4.14 mg, vitamin B6 4.53 mg, vitamin B12 6 μg, folic acid 414 μg, mg, vitamin B7 69 μg, vitamin PP 46 mg
Infusion route	Angocath
Daily infusion duration	13 h
Full infusion rate	150 mL/h
Total duration of PPN	7 days
Monitoring	Daily inspection of the venous catheter entry site, dressing change every 5 days, and use of devices dedicated to the aseptic management of the access
Refeeding syndrome monitoring	Daily monitoring of anthropometric, clinical, and biochemical parameters.
Catheter-related complications	none

Abbreviations: PPN, peripheral parenteral nutrition; SMOF, Soybean oil, Medium-chain triglycerides, Olive oil, Fish oil.

**Table 3:** EN and PPN administration protocol for transition to full EN.

Composition	Value
Kcal	1 Kcal/mL
Administration route	PEG
Day of administration	Value
First day	500 mL (administration rate 50 mL/hour) plus 700 mL of PPN
Second day	750 mL (administration rate 55 mL/hour) plus 500 mL of PPN
Third day	1000 mL of EN (administration rate 60 mL/hour).

Abbreviations: PEG, percutaneous endoscopic gastrostomy; PPN, peripheral parenteral nutrition; EN, enteral nutrition.

**Table 5:** Clinical parameters: admission.

Parameter/Examination	Results
Charlson Comorbidity Index	1
Karnofsky Performance Status	80%
Height (cm)	159
CONUT Index	2
NRS-2002	4
MUST	2
Glim criteria	Positive
Heart rate (bpm)	58
Blood pressure (mmHg)	80/58
Oxygen saturation (SpO_2_)	99% on ambient air
Temperature	Afebrile
Spontaneous urination	Adequate
Bowel function	Alternating
General Physical Examination	Hypotrophic muscular system across all body regions; dehydrated skin and mucous membranes.
Respiratory Examination	Diffuse vesicular breath sounds throughout the pulmonary fields; no pathological adventitious sounds.
Abdominal Examination	Abdomen flat, soft, non-tender to superficial palpation; mild diffuse tenderness on deep palpation; tympanic on percussion; hypochondrial organs not palpable.
Examination of the lower limbs	No signs of active deep vein thrombosis; mild pitting edema in dependent areas.
Neurological examination:	No appreciable cranial nerve deficits; no sensory-motor deficits in any of the four limbs.

Abbreviations: CONUT, COntrolling NUtritional Status; Glim, Global Leadership Initiative on Malnutrition; MUST, Malnutrition Universal Screening Tool; NRS, Nutritional Risk Screening.

**Table 6:** Nutritional parameters and bioelectrical impedance analysis: admission (the definitions and physiological interpretation of the bioimpedance parameters are provided in the Appendix A).

Parameter	Values
Body Weight (kg)	36
Body Mass index	14.2
Resistance (Ohms, Ω)	450
Reactance (Ohms, Ω)	29.1
Phase Angle (Degrees °)	3.7
Lean Body Mass (kg)	34.1
Extracellular (%)	+15.3
Fat Mass (kg)	1.9
Basal Metabolic Rate (kcal/day)	1138.3

**Table 7:** Abnormal blood chemistry tests: admission.

Parameter	Values	Normal Range
Albumin (g/dL)	3.2	3.4–4.8
Zinc (γ/dL)	54	68–107
Ferritin (ng/mL)	6	12–290
Vitamin D (ng/mL)	11	31–100
Amylase (U/L)	157	30–118
ALT (GPT) (U/L)	48	<49
γGT (U/L)	43	<38
LDH (U/L)	266	<250

Abbreviations: ALT, alanine aminotransferase; LDH, lactate dehydrogenase; γGT, γ-glutamyl transferase.

**Table 8:** Peripheral parenteral nutrition protocol.

Parameter	Value
Body weight	36 kg
Body mass index	14.2 kg/m^2^
Indication for PPN	Severe protein–energy malnutrition associated with anorexia nervosa and intolerance to nasogastric feeding
PPN formulation	Three-compartment peripheral parenteral nutrition admixture
Energy provided (full regimen)	1000 kcal/day
Energy	27.8 kcal/kg/day
Initial energy delivery	Approximately 500 kcal/day (50% of target)
Initial energy	Approximately 13.9 kcal/kg/day
Amino acids	46 g/day
Protein delivery	1.28 g/kg/day
Carbohydrate	100 g glucose/day
Total infusion volume	1448 mL/day
Initial infusion volume	Approximately 724 mL/day
Osmolarity	Approximately 850 mOsm/L (peripheral-compatible formulation)
Lipid formulation	Fourth-generation lipid emulsion containing soybean oil, medium-chain triglycerides, olive oil, and fish oil
Electrolytes	Standard electrolyte composition (sodium, potassium, calcium, magnesium, phosphate, chloride, and acetate)
Micronutrient supplementation	Intravenous vitamins, trace elements, and prophylactic thiamine
Infusion route	Peripheral venous catheter
Daily infusion duration	24 h continuous infusion
Initial infusion rate	Approximately 30 mL/h
Full infusion rate	Approximately 60 mL/h
Total duration of PPN	10 days
Initial infusion rate	Approximately 30 mL/h
Full infusion rate	Approximately 60 mL/h
Monitoring	Daily clinical assessment; serum phosphate, magnesium, potassium, glucose, hydration status, vital signs, and liver and renal function
Refeeding syndrome monitoring	Daily biochemical monitoring during gradual caloric advancement
Catheter-related complications	None observed

Abbreviations. PPN, peripheral parenteral nutrition.

**Table 9:** Sequence of the nutritional treatment.

1	2	3
5 days of mixed-feeding PPN + ONS in hospital care	5 days of mixed-feeding PPN + ONS in day-hospital care	Oral feeding Personalized dietary plan + ONS in home care

Abbreviations: PPN, peripheral parenteral nutrition; ONS, oral nutritional supplementation.

**Table 10:** Laboratory findings at admission.

Parameter	Value	Normal Range
Hemoglobin (g/dL)	10.8	12.2–15.5
White blood cells (n/µL)	17,800	4000–10,000
Platelets (n/µL)	55,000	150,000–400,000
INR	2.5	0.8–1.2
Fibrinogen (mg/dL)	610	160–350
AST (GOT) (U/L)	285	10–35
ALT (GPT) (U/L)	210	10–35
Alkaline phosphatase (U/L)	380	40–130
γGT (U/L)	420	6–42
Total bilirubin (mg/dL)	3.1	0.15–1.2
Direct bilirubin (mg/dL)	2.4	0.08–0.3
Serum amylase (U/L)	1180	28–100
Lipase (U/L)	1420	13–60
LDH (U/L)	680	135–214
Total proteins	4.8	6.4–8.3
Albumin (g/dL)	2.5	3.5–5.2
Prealbumin (mg/dL)	10	20–40
Total cholesterol (mg/dL)	90	<190
CRP (mg/dL)	18	<0.5
PCT (ng/mL)	6.2	<0.09
Sodium (mmol/L)	130	136–148
Potassium (mmol/L)	4.8	3.5–5.1
Calcium (mg/dL)	7.6	8.60–10.0
Phosphorus (mg/dL)	2.2	2.5–4.5
Magnesium (mg/dL)	1.3	1.6–2.6
Creatinine (mg/dL)	1.3	0.5–1.0
Glucose (mg/dL)	125	74–106
Pseudocholinesterase (U/L)	3200	5300–12,900

Abbreviations: INR, International Normalized Ratio; ALT, alanine aminotransferase; AST (GOT), aspartate aminotransferase; γGT, γ-glutamyl transferase; LDH, lactate dehydrogenase; CRP, C-reactive protein; PCT (ng/mL), procalcitonin.

**Table 11:** Peripheral parenteral nutrition protocol.

Parameter	Value
Body weight	60.7 kg
Body Mass Index	21.4 kg/m^2^
Indication for PPN	Abdominal pain. Acute pancreatitis. Technical difficulties in placing nasojejunal tube
PPN formulation	SMOF 1904 mL
Energy provided (full regimen)	1300 kcal
Amino acids	68 g
Carbohydrate	135 g
Total infusion volume	1904 mL
Osmolarity	850 mosm/L
Lipid formulation	54 gr (30% soy oil, 30% medium-chain triglycerides, 25% olive oil, and 15% fish oil) providing ω-3 fatty acids and yielding an n-6:n-3 ratio of 2.5:1.
Electrolytes	Na 48 mmol, K 36 mmol, Mg 6.0 mmol, Ca 3.6 mmol, P 14.5 mmol
Micronutrient supplementation	Cr 0.02 mcmol, Cu 20 mcmol, Fe 20 μmol, Mn 5 mcmol, I 1 μmol, Fl 50 μmol, Mo 0.2 μmol, Se 0.6 μmol, Zn 100 μmol. Vitamin A 3.500 UI, vitamin D3 220 UI, vitamin E 11.2 UI, vitamin C 125 mg, vitamin B1 3.51 mg, Vitamin B2 4.14 mg, vitamin B6 4.53 mg, Vitamin B12 6 μg, folic acid 414 μg, vitamin B5 17.25 mg, vitamin B7 69 μg, Vitamin PP 46 mg
Infusion route	Angiocath
Daily infusion duration	13 h
Full infusion rate	150 mL/h
Total duration of PPN	7 days
Monitoring	Daily inspection of the venous catheter entry site, dressing change every 5 days, and the use of devices dedicated to the aseptic management of the access.
Refeeding syndrome monitoring	Daily monitoring of anthropometric, clinical, and biochemical parameters.
Catheter-related complications	None observed

Abbreviations: PPN, peripheral parenteral nutrition; SMOF, Soybean oil, Medium-chain triglycerides, Olive oil, Fish oil.

**Table 12:** Sequence of the nutritional treatment.

1	2	3
8 days of exclusive PPN	3 days of mixed-feeding phase PPN + ON	Full ON

Abbreviations: PPN, peripheral parenteral nutrition; ON, oral nutrition.

**Table 13:** Trends of coagulation profile and inflammatory markers.

Parameter	Admission	Day 3	Day 8	Day 11	Normal Range
INR	2.5	2.1	1.6	1.3	0.8–1.2
CRP (mg/dL)	18	10.5	3.8	0.9	<0.5
PCT (ng/mL)	6.2	3.5	0.8	0.2	<0.1
Total proteins (g/dL)	4.8	5.2	6.1	6.8	6.0–8.0

Abbreviations: INR, International Normalized Ratio; CRP, C-reactive protein; PCT, procalcitonin.

**Table 14:** Trends of electrolyte levels.

Parameter	Admission	Day 3	Day 8	Day 11	Normal Range
Sodium (mmol/L)	130	135	138	140	135–145
Potassium (mmol/L)	4.8	4.5	4.2	4.1	3.5–5.0
Calcium (mg/dL)	7.6	7.9	8.6	8.9	8.5–10.5
Phosphorus (mg/dL)	2.2	2.4	3.1	3.4	3.5–5.1
Magnesium (mg/dL)	1.3	1.5	1.7	1.9	1.6–2.4

**Table 15:** Trends of renal function and metabolic profile.

Parameter	Admission	Day 3	Day 8	Day 11	Normal Range
Creatinine (mg/dL)	1.3	1.1	0.9	0.8	0.6–1.2
Glucose (mg/dL)	125	140	130	110	70–110
Total cholesterol (mg/dL)	90	100	120	135	130–200

**Table 16:** Trends of pancreatic enzymes and liver biochemistry.

Parameter	Admission	Day 3	Day 8	Day 11	Normal Range
Serum amylase (U/L)	1180	780	340	120	25–125
Lipase (U/L)	1420	910	380	160	13–60
AST (U/L)	285	190	95	48	10–40
ALT (U/L)	210	145	82	40	10–40
γGT (U/L)	420	350	220	160	10–50
ALP (U/L)	380	320	240	180	40–130

Abbreviations: AST, aspartate aminotransferase; ALT, alanine aminotransferase; γGT, gamma-glutamyl transferase; ALP, alkaline phosphatase.

## Data Availability

No new data were created or analyzed in this study. Data sharing is not applicable to this article.

## Supplementary Materials

The following supporting information can be downloaded at https://www.mdpi.com/article/10.3390/healthcare14152413/s1, Checklist CARE.

## Abbreviations

AN	Anorexia Nervosa
ALP	Alkaline Phosphatase
ALT (GPT)	Alanine Aminotransferase
AP	Acute Pancreatitis
AST (GOT)	Aspartate Aminotransferase
CONUT	COntrolling NUtritional Status
CPN	Central Parenteral Nutrition
EN	Enteral Nutrition
ESPEN	European Society for Clinical Nutrition and Metabolism
GLIM	Global Leadership Initiative on Malnutrition
γGT	γ-Glutamyl Transferase
INR	International Normalized Ratio
LDH	Lactate Dehydrogenase
MUST	Malnutrition Universal Screening Tool; NRS
NRS	Nutritional Risk Screening
ON	Oral Nutrition
ONS	Oral Nutritional Supplementation
PEG	Percutaneous Endoscopic Gastrostomy.
PN	Parenteral Nutrition
PPN	Peripheral Parenteral Nutrition
PCT	Procalcitonin
CRP	C-Reactive Protein
SAP	Severe Acute Pancreatitis

## Appendix A. Description of the Bioelectrical Impedance Parameters [32,33,34,35,36]

**Parameter**	**Description**	**Measurement Unit**
Basal Metabolic Rate	Minimum energy needed to sustain vital physiological functions at rest.	Kilocalories/day (kcal/day)
Extracellular Water	Volume of water outside the cells, including plasma and interstitial fluid.	Percentage (%)
Fat Mass	Total amount of adipose tissue, including essential and storage fat.	Kilograms (kg, percentage of total body mass)
Lean Body Mass	Total body mass excluding fat, including muscle, organs, bone, and body water.	Kilograms (kg);
Phase Angle	Phase shift between current and voltage derived from resistance and reactance; marker of nutritional status and cellular integrity. High values indicate better membrane integrity; low values may reflect malnutrition, inflammation, or chronic disease.	Degrees (°)
Reactance	Indicator of the dielectric properties of cell membranes and active cell mass. Low values may suggest cellular impairment or malnutrition.	Ohms (Ω)
Resistance	Body’s opposition to electrical current; mainly determined by total body water. High values may indicate dehydration or low lean mass.	Ohms (Ω)
