# Supplementary material for: The Use of Peripheral Parenteral Nutrition in Hospitalized Patients: A Clinical Case Series
Source: Healthcare (Basel). 2026 Aug 5;14(15):2413. doi: 10.3390/healthcare14152413 (PMC13464783; doi:10.3390/healthcare14152413)
Supplement: Supplementary file 1 [file healthcare-14-02413-s001.zip › healthcare-4363998-supplementary.pdf]

Checklist CARE			
**Topic**	**Item**	**Checklist item description**	**Location**
**Title**	**1**	The diagnosis or intervention of primary focus followed by the words "case series".	Title
**Keywords**	**2**	Two to five key words that identify diagnoses or interventions in this case report, including "case series".	Keywords section
**Abstract**	**3a**	Introduction: What is unique about this case and what does it add to the scientific literature?	Lines 10 – 13 of abstract introduction
	**3b**	Main symptoms and/or important clinical findings.	Lines 6 to 10 of abstract Introduction.
	**3c**	The main diagnoses, therapeutic interventions, and outcomes	- Lines 9 - 10 of abstract introduction.<br>-Abstract: Results.
	**3d**	Conclusion—What is the main "take-away" lesson(s) from this case?	Abstract Conclusions.
**Introduction**	**4**	One or two paragraphs summarizing why this case series is unique (may include references).	Section1, Paragraph 5
**Patient Information**	**5a**	De-identified patient specific information.	**Case1**. Section 2.1.1, Paragraph 1<br>**Case 2**. Section 2.2.1 Paragraphs 1-4<br>**Case 3.** Section 2.3.1, Paragraphs 1-3
	**5b**	Primary concerns and symptoms of the patient.	**Case 1.** Section 2.1,Figure 1 – Flow chart.<br>Section 2.1.1, Paragraph 1<br>**Case 2**.Section 2.2, Figure 4- Flow chart<br>Section 2.2.1,Paragraphs 1,<br>**Case 3.** Section 2.3, Figure 6 – Flow chart<br>Section 2.3.1, Paragraphs 1
	**5c**	Medical, family, and psycho-social history, including relevant genetic information.	**Case 1.** Section 2.1.1 Paragraph 1<br>**Case 2**. Section 2.2.1, Paragraphs 12-4<br>**Case 3**. Section 2.3.1, Paragraphs 1-3
	**5d**	Relevant past interventions with outcomes.	**Case 1**. Not available<br>**Case 2**. Section 2.2.1, Paragraphs 4<br>**Case 3**. Section 2.3.1 Paragraph 1
**Clinical Findings**	**6**	Relevant signs and symptoms at presentation.	**Case 1**. Section 2.1 - Flow chart, Section 2.1.1, Paragraphs 2-4<br>**Case 2**. Section 2.2 – flowchart Section 2.2.1, Paragraphs 1,5,6

Checklist CARE			
**Topic**	**Item**	**Checklist item description**	**Location**
			**Case 3**. Section 2.3 – Flowchart. Section 2.3.1, paragraphs 1
**Timeline**	**7**	Historical and current information from this episode of care, organized as a timeline.	**Case 1**. Section 2.1, Figure 1 – Flow chart. Section 2.1.1 Table 4<br>**Case 2**. Section 2.2, Figure 4 - Flow chart. Section 2.2.1, Table 9<br>**Case 3**. Section 2.3, Figure 6- Flow chart. Section 2.3.1, Table 12
**Diagnostic Assessment**	**8a**	Diagnostic testing (such as PE, laboratory testing, imaging, surveys).	**Case 1**. Section 2.1.1, Paragraphs 3-5<br>**Case 2**. Section 2.2.1, Tables 5-7<br>**Case 3**. Section 2.3.1, Paragraphs 4, 5, Table 10
	**8b**	Diagnostic challenges (such as access to testing, financial, or cultural).	**Case 1**. Section 2.1,1, paragraph 11<br>**Case 2**. Section 2.2.1,, Paragraphs 7,8.<br>Section 2.2.2, Paragraph 11, Section 2.2.3, paragraph 10<br>**Case 3**. Not applicable
	**8c**	Diagnosis (including other diagnoses considered).	**Case 1**. Section 2.1 - Flow chart. Section 2.1.2, paragraph 5<br>**Case 2**. Section 2.2.- Flow chart Section 2.2.1, paragraph 7<br>**Case 3**. Section 2.3 - Flow chart. Section 2.3.1, paragraph 6.
	**8d**	Prognosis (such as staging in oncology) where applicable.	Not applicable
**Therapeutic Interventions**	**9a**	Types of therapeutic intervention (such as pharmacologic, surgical, preventive, self-care).	**All cases**: Abstract, lines 6-8<br>**Case 1**. Section 2.1 – Flowchart. Section 2.1.1, paragraph 7.<br>**Case 2.** Section 2.1- Flow chart. Section 2.2.1, paragraph 8-10<br>**Case 3**. Paragraphs 8, 9.
	**9b**	Administration of therapeutic intervention (such as dosage, strength, duration).	,Case 1. Section 2.1.1, paragraph 6-10. Tables 2- 4.<br>Case 2.. Section 2.2.1, paragraphs 9,10. Tables 8, 9.<br>Case 3.. Section 3.3.1, Paragraphs 7-9. Tabes 11, 12.

Checklist CARE			
**Topic**	**Item**	**Checklist item description**	**Location**
	**9c**	Changes in therapeutic intervention (with rationale).	Not applicable
**Follow-up and Outcomes**	**10a**	Clinician and patient-assessed outcomes (if available).	**Case 1**. Not available<br>**Case 2**. Not available<br>**Case 3**. Not available
	**10b**	Important follow-up diagnostic and other test results.	**Case 1**. Figures 2, 3<br>**Case 2**. Section 2.2.1, Figures 5a,b,c.<br>**Case 3**. Not available.
	**10c**	Intervention adherence and tolerability (How was this assessed?).	**Case** 1. Section 2.1.1, Paragraph 11, Table 2.<br>**Case 2**. Section 2.2.1, paragraphs 11, 13, 15<br>**Case 3**. Section 2.3.1, paragraph 12, Table X.
	**10d**	Adverse and unanticipated events.	**Case 1**. Section 2.1.1, Paragraph 11, Table 2.<br>**Case 2**. Section 2.2.1, paragraphs 11, 15<br>**Case 3.** Section 2.3.1, paragraph 11, Table 11.
**Discussion**	**11a**	A scientific discussion of the strengths AND limitations associated with this case report.	Section 3, Paragraph 14,15
	**11b**	Discussion of the relevant medical literature with references.	**Case 1.** Section 2.1.2, Section paragraph 1,2.<br>Section 3, paragraph 4.<br>**Case 2.** Section 2.2.2, Paragraph 1-7<br>Section 3, paragraph 5<br>**Case 3**. Section 2.3.2, Paragraphs 1-9<br>Section 3, paragraph 6<br>**All cases**: Section 1 and 3
	**11c**	The scientific rationale for any conclusions (including assessment of possible causes).	**Case 1**. Section 2.1.2, paragraphs 1-3, 5.<br>**Section 3**, paragraph 4<br>**Case 2**. Section 2.2.2, paragraphs 1-3.<br>Section 3, paragraph 5.<br>**Case 3**. Section 2.3.2, paragraphs 1-7<br>Section 3, Paragraph 1,2<br>**All cases.** Section 3
	**11d**	The primary "take-away" lessons of this case report (without references) in a one-paragraph conclusion.	Section 4.

Checklist CARE			
**Topic**	**Item**	**Checklist item description**	**Location**
**Patient Perspective**	**12**	The patient should share their perspective in one to two paragraphs on the treatment(s) they received.	**Case 1.** Section 2.1.1, Paragraph 11<br>**Case 2**. Section 2.2.1, Paragraph 15<br>**Case 3**. Section 2.3.1, Paragraph 11.
**Informed Consent**	**13**	Did the patient give informed consent? Please provide if requested.	Yes ☐ No ☑<br>Section: Informed Consent Statement
